# Factors associated with unsatisfactory cosmetic results in oncoplastic surgery

**DOI:** 10.3389/fonc.2023.1071127

**Published:** 2023-07-24

**Authors:** Idam de Oliveira-Junior, René Aloísio da Costa Vieira, Gabriele Biller, Almir José Sarri, Fabíola Cristina Brandini da Silva, Eliana Aguiar Petri Nahás

**Affiliations:** ^1^ Postgraduate Program of Tocogynecology, Botucatu Medical School, Sao Paulo State University, UNESP, Sao Paulo, Brazil; ^2^ Postgraduate Program of Oncology, Barretos Cancer Hospital, Sao Paulo, Brazil; ^3^ Nucleous of Mastology, Barretos Cancer Hospital, Sao Paulo, Brazil; ^4^ Faculty of Health Sciences of Barretos Dr. Paulo Prata (FACISB) School of Medicine, Sao Paulo, Brazil

**Keywords:** breast cancer, conservative surgery, breast-conserving surgery, oncoplastic surgery, cosmesis

## Abstract

**Introduction:**

Oncoplastic surgery (OS) has expanded the indications for breast-conserving surgery associated with an adequate aesthetic result. However, few studies have described the factors associated with unsatisfactory cosmetic outcomes from this surgical modality.

**Materials and methods:**

This is a cross-sectional prospective study that included patients undergoing breast-conserving surgery (BCS) with or without OS. The patients self-evaluated the cosmetic results of the breasts posttreatment and had them photographed. The photos were analyzed by BCCT.core. Individual and treatment factors (local and systemic) for all patients were evaluated. These factors were dichotomized according to the use of OS and to the cosmetic result (satisfactory and unsatisfactory). Categorical variables were tested for association with surgical outcome using the chi-square test while numerical variables using the Mann−Whitney U test. Variables with p <0,2 were selected for multivariate analysis.

**Results:**

Of the 300 patients evaluated, 72 (24,0%) underwent OS. According to the patient self-evaluations, an unsatisfactory cosmetic result from OS was significantly associated with younger age at diagnosis, higher body mass index (BMI) at the time of evaluation, larger tumor size and greater weight of the surgical specimen. According to the BCCT.core, only the laterality of the tumor (left) was significantly associated with an unsatisfactory cosmetic result. In logistic regression, considering OS as a control variable, the risk of an unsatisfactory outcome according to patient self-evaluation was related to the tumor ≥ T2 odds ratio (OR) 1,85 (1,027-3,34) and age at diagnosis < 40 [OR 5,0 (1,84-13,95)]. However, according to the software, the variables were associated with an increased risk of an unsatisfactory outcome were the time interval between surgery and evaluation [OR 1,27 (1,16-1,39)], the presence of lymphedema [OR 2,97 (1,36-6,46)], surgical wound infection [OR 3,6 (1,22-11,16)], tumor location on the left side [OR 3,06 (1,69-5,53)], overweight [OR 2,93 (1,48-5,8)] and obesity [OR 2,52 (1,2-5,31)].

**Conclusion:**

There is no standard methodology for breast cosmesis evaluation, which influences the factors associated with unsatisfactory results. Younger patients and those with increased BMI, left breast cancer and extensive resections tend to present with unsatisfactory cosmetic results when OS is performed.

## Introduction

The outcome of the surgical treatment of breast cancer extends beyond purely oncological issues (1–3). The unsatisfactory cosmetic results of breast conservative surgery motivated the development of oncoplastic surgery (OS), which, by incorporating concepts and techniques of plastic surgery in the treatment of breast cancer, has allowed an increase in the number of breast conservation indications as well as better cosmetic results (4–6). However, OS is highly technically variable, which involves from small parenchyma remodeling to complex resections, making it difficult to judge the results and limiting oncological and cosmetic comparisons (2, 7, 8).

Cosmetic analysis after conservative breast treatment can be performed through subjective methods, which take into account the self-evaluation of the patient or the analysis of the health professionals involved in the treatment, and objective methods, which consider the measurement of asymmetry between the treated *versus* untreated breast. The lack of standards in the evaluation of these results and the low agreement between them directly influence the reproducibility and validity of the methods (9–13). In this sense, the BCCT.core (*Breast Cancer Conservative Treatment Cosmetic Results*) software, which employs algorithms for calculating breast symmetry and yields highly correlated results calibrated by specialists, was established to contend with these problems (14, 15).

Unsurprisingly, breast conserving-surgery (BCS) typically results in varying degrees of breast asymmetry, which can negatively affect the quality of life of women (16). The main factors associated with unsatisfactory cosmetic results after BCS classical surgery (CS) include high body mass index (BMI) and tumor size, advanced age, tumor location (medial, central or lower quadrants), reexcision, small breast volume, heterogeneity of the radiation dose and resection of the breast parenchyma greater than 100 cm^3^ (17–19). However, few studies have investigated the factors that can influence the outcomes of OS. Therefore, it necessary to investigate these factors to better understand them, to optimize the information delivered to the patient, and prevent their onset. Such a study would also assist in monitoring the cosmetic results over time and their relationship with the patient profile, surgical technique and adjuvant therapies.

## Materials and methods

This was a prospective cross-sectional study approved by the Research Ethics Committee under number 782/2014 with support from FAPESP (2014/08197-0) that randomly included patients followed up at the Mastology and Breast Reconstruction outpatient clinic of the Barretos Cancer Hospital who underwent BCS (CS or OS) for the treatment of breast cancer.

Patients who had completed radiotherapy at least one year prior, without metastatic disease and/or locoregional recurrence and who signed the informed consent form were included in the study. Patients with bilateral breast cancer, male patients and those with cognitive limitations for cosmetic self-assessment were excluded.

The patients were photographed in a standardized manner (1 meter distance with a point marked on the sternal furcula and another 20 cm below, at the sternal level, for distance calibration) and self-assessed the cosmetic result of the breast (excellent, good, reasonable, or poor). The photographs were analyzed prospectively, cross-sectionally and blindly using BCCT.core software, which provides results on a 4-point scale (1-excellent, 2-good, 3-fair, 4-poor). For patients with no areola, a central point on the breast was marked when possible. This methodology was previously published (12, 13).

In the cosmetic evaluation, classifications of excellent and good, both by the patient and by BCCT.core, were considered satisfactory. Conversely, evaluations classified as reasonable/fair and poor were categorized as unsatisfactory cosmetic results.

Next, breast cosmesis categorized as satisfactory and unsatisfactory were evaluated by the patient and the BCCT.core software and correlated with the patient’s personal and oncological history, obtained retrospectively from the medical records.

### Statistical analysis

The data were initially analyzed for all patients undergoing conservative breast treatment (classic and with oncoplasty). Subsequently, they were dichotomized according to the use of breast oncoplasty and according to the cosmetic result (satisfactory and unsatisfactory). For the categorical variables, the frequencies (absolute and relative) are reported. Numerical variables are reported as the mean, median and standard deviation.

The associations between surgical outcome and categorical variables were performed using the chi-square test; those for continuous variables were calculated with the Mann−Whitney test.

The variables with a descriptive p level<0,20 in the analysis of all cases and OS group were selected for multivariate analysis. An adjusted logistic regression model was derived to calculate the odds ratio (OR) and its respective 95% confidence interval (95% CI) for an unsatisfactory cosmetic result. Because it is important for analysis, oncoplastic surgery (present vs. absent) was considered as a control variable in all cases. The analyses were performed using IBM-SPSS software version 27.0, and a 5% significance level was adopted.

## Results

A total of 300 patients were evaluated, as described in a previous study, for validation of the BCTOS questionnaire (12). Of these, 298 had their photographs evaluated by BCCT.core, and 297 performed a breast self-assessment. A total of 228 (76,0%) patients underwent CS, and 72 (24,0%) underwent OS; of these, 37 (51,4%) underwent contralateral symmetrization surgery. The mean follow-up time from the first medical evaluation to participation in the study was 7,4 years (1,2-20,6; standard deviation 4,3). Among patients who underwent OS, 73,6% self-assessed a satisfactory cosmetic result, and 26,4% considered the outcome unsatisfactory; according to the software analysis, 29,2% and 70,8% of the outcomes were satisfactory and unsatisfactory, respectively.

In the patient self-evaluations, across all patients (**Supplementary Tables 1**, **2**), factors such as younger age at diagnosis and at the time of evaluation (gross and by age group), larger tumors (either categorically according to the T stage in the TNM classification or numerically), the use of radiation therapy boost and a longer time interval between surgery and evaluation and between the end of radiotherapy and evaluation were significantly associated with unsatisfactory cosmetic results.

Among patients who underwent classical conservative treatment (quadrantectomy), according to their self-assessment, factors such as younger age at diagnosis (categorical or numerical), variability in radiotherapy dose, the use of boost and a longer time interval between surgery and evaluation and between the end of radiotherapy and evaluation were significantly associated with unsatisfactory cosmetic results. However, among those treated with OS, an unsatisfactory result was significantly associated with younger age at diagnosis, higher BMI at the time of assessment (categorical or numerical), larger tumor size and greater weight of the surgical specimen. In the logistic regression for overall unsatisfactory cosmetic outcome (**Table 1**), in the patient selfevaluation, a significantly increased risk was observed for tumors ≥ T2 odds ration (OR) 1,86 (1,035–3,35)] and age <40 years [OR = 5,1 (1,86–14,01)]. When using OS variable as a control (**Table 1**), tumor ≥ T2 [OR =1,85 (1,027–3,34)] and age <40 years [OR = 5,1 (1,86-14,01)]. When using OS variable as a control (**Table 1**), tumor ≥ T2 odds ratio (OR) 1,85 (1,027-3,34) and age at diagnosis < 40 years [OR 5,0 (1,84-13,95)] was associated with increased risk of an unsatisfactory outcome. In the OS group (**Table 2**), only tumor size was associated with an increased risk of an unsatisfactory result [≥ T2 OR = 7,205 (1,403 – 37,017)].

**Table 1:** Logistic regression for unsatisfactory cosmetic results in all cases according to different criteria for evaluation.

Type of evaluation	Category	Variable	OR	p variable	p group
**Patient’s evaluation**	**TNM stage - T**	Tis and T1	reference		0,038
		≥ T2	1,86 (1,035 – 3,35)		
	**Age at diagnosis (years)**	≥ 60	reference		0,003
		50 – 59	1,84 (0,762 – 4,45)	0,175	
		40 – 49	1,2 (0,49 – 3,04)	0,667	
		< 40	5,1 (1,86 – 14,01)	0,002	
**Patient’s evaluation**	**Oncoplastic surgery**	Absent	reference		0,817
**OS control variable**		Present	1,09 (0,52 – 2,25)		
	**TNM stage - T**	Tis and T1	reference		0,04
		≥ T2	1,85 (1,027 – 3,34)		
	**Age at diagnosis (years)**	≥ 60	reference		0,003
		50 – 59	1,83 (0,76 – 4,44)	0,177	
		40 – 49	1,2 (0,48 – 3,03)	0,679	
		< 40	5,0 (1,84 – 13,95)	0,002	
**BCCT.core software**	**Weight of the surgical specimen (g)**	Continuous	1,004 (1,001 – 1,008)		0,01
	**Time between surgery and evaluation (years)**	Continuous	1,26 (1,15 – 1,38)		<0,001
	**Lymphedema**	Absent	reference		0,018
		Present	2,54 (1,17 – 5,53)		
	**Surgical wound infection**	Absent	reference		0,04
		Present	3,06 (1,04 – 8,99)		
	**Tumor side**	Right	reference		<0,001
		Left	2,96 (1,64 – 5,34)		
	**BMI at diagnosis (kg/m^2^)**	<25	reference		0,02
		25 – 29,9	2,57 (1,3 – 5,0)	0,006	
		≥ 30	1,85 (0,86 – 4,01)	0,115	
**BCCT.core software**	**Oncoplastic surgery**	Absent	reference		0,065
**OS control variable**		Present	2,13 (0,95 – 4,76)		
	**Time between surgery and evaluation (years)**	Continuous	1,27 (1,16 – 1,39)		<0,001
	**Distance from the surgical margin**	Continuous	1,68 (0,97 – 2,92)		0,062
	**Lymphedema**	Absent	reference		0,006
		Present	2,97 (1,36 – 6,46)		
	**Surgical wound infection**	Absent	reference		0,02
		Present	3,6 (1,22 – 11,16)		
	**Tumor side**	Right	reference		<0,001
		Left	3,06 (1,69 – 5,53)		
	**BMI at diagnosis (kg/m^2^)**	<25	reference		0,005
		25 – 29,9	2,93 (1,48 – 5,8)	0,002	
		≥ 30	2,52 (1,2 – 5,31)	0,01	

OS – oncoplastic surgery/BMI – body mass index.

**Table 2:** Logistic regression for unsatisfactory cosmetic results in OS group according to different criteria for evaluation.

Type of evaluation	Category	Variable	OR	p group
Patient’s Assessment	TNM stage - T	Tis and T1	reference	0,018
		≥ T2	7,205 (1,403 – 37,017)	
BCCT.core Software	Tumor side	Right	reference	0,01
		Left	4,21 (1,43 – 15,27)	

In the analysis of the BCCT.core software (**Supplementary Tables 3**, **4**) across all patients, a higher BMI (numerical and categorical) at diagnosis and at the time of evaluation, tumors with a higher T stage (TNM), a tumor of the left side, axillary lymphadenectomy, chemotherapy, radiotherapy dose of 28 x 180 cGy, presence of lymphedema (evaluated based on the water displacement methodology, considered present when the difference between the upper limbs had a value greater than or equal to 200 milliliters (12)), greater weight of the surgical specimen, greater distance from the surgical margins and longer time interval between surgery and evaluation and between the end of radiotherapy and evaluation were significantly associated with unsatisfactory cosmetic results. Conversely, among patients undergoing CS, higher BMI at diagnosis (categorical and numerical) and at the time of evaluation, lower patient educational level, tumor on the left side, axillary lymphadenectomy and chemotherapy, radiotherapy dose of 28 x 180 cGy, higher weight of the specimen and distance from the surgical margins and a longer time interval between surgery and evaluation and between the end of radiotherapy and evaluation were significantly associated with unsatisfactory cosmetic results. However, among those treated with OS, only tumors on the left side were related to an unsatisfactory cosmetic result. In the logistic regression analysis for unsatisfactory cosmetic outcomes across all patients (**Table 1**), there was an increased risk for the weight of the surgical specimen [OR = 1,004 (1,001-1,008)], the time interval between surgery and evaluation [OR = 1,26 (1,15-1,38)], the presence of lymphedema [OR = 2,54 (1,17-5,53)], the occurrence of surgical wound infection [OR 3,06 (1,04-8,99)], tumor on the left side [OR 2,96 (1,64-5,34)] and overweight at diagnosis [OR = 2,57 (1,3-5,0)]. Using OS as a control variable in the logistic regression (**Table 1**), an increased risk of unsatisfactory results was observed in the time interval between surgery and evaluation [OR 1,27 (1,16-1,39)], the presence of lymphedema [OR 2,97 (1,36-6,46)], surgical wound infection [OR 3,6 (1,22-11,16)], tumor location on the left side [OR 3,06 (1,69-5,53)], overweight [OR 2,93 (1,48-5,8)] and obesity [OR 2,52 (1,2-5,31)]. In the logistic regression in the OS group (**Table 2**), only the laterality of the tumor influenced the unsatisfactory result [left side OR 4,21 (1,43-15,27)].

## Discussion

OS includes oncological treatment *versus* adequacy of the residual breast volume, whether or not associated with symmetrization of the contralateral breast. In addition to expertise, in choosing the technique, the surgeon must consider the characteristics of both the tumor and the breast and understand the expectations and frustrations of the patient, remaining cognizant to the fact that there is no single formula to solve the difficulties imposed by the tumor and that OS is not a guarantee of good cosmetic results (2, 20).

Our study, which included only patients undergoing BCS, sought to evaluate the factors associated with an unsatisfactory cosmetic outcome in OS, both according to the patient and through a previously consolidated and reproducible objective methodology *via* BCCT.core. The correlation among cosmetic evaluations of breast surgery is low among different methodologies (16, 21), as are the factors that influence unsatisfactory results. In the patient self-assessment, younger women with higher BMI at the time of evaluation, with larger tumors and heavier surgical specimen tended to present with unsatisfactory breast results. However, according to the software, only the laterality of the tumor influenced these results. We observed a non-significant increase in the unsatisfactory result in the logistic regression using OS as a control variable. However, this fact may be related to the presence of larger tumors, younger patients (potentially questioning) and the small number of contralateral symmetrization in the oncoplastic surgery group.

Understanding these factors allows sharing of the decision-making process with the patient and guidance of training programs for breast surgeons. Indeed, the aesthetic result of breast surgery is closely related to the woman’s body self-image, sexual function and quality of life. In addition, because breast cancer survival has been a reality and patients end up experiencing greater treatment morbidity over the years, the aesthetic outcome of the breast has become one of the pillars of cancer treatment, with a priority of patient satisfaction. Thus, the results reported by the patients are a current issue that deserves to be discussed and addressed, as in the present study (21, 22).

In judging the cosmetic result of the breasts after conservative treatment, it is necessary to take into account considerations, often not found in the literature, that influence the factors related to unsatisfactory outcomes. The first corresponds to the time in which the evaluation is performed. Over the years, the woman presents with body changes, sequelae of systemic therapy, and the chronic and progressive effect of radiotherapy, which accentuates the asymmetry in breast size and shape and causes the deceleration of natural breast ptosis and asymmetry in the position of the areolo-papillary complex and skin color (22, 23). Second, we emphasize that the analysis of cosmetic results requires a gold standard and that current methods can show wide variability. Likewise, the literature is heterogeneous with regard to the predisposing factors for unsatisfactory breast cosmesis, primarily due to differences in the studies with regard to the design, size and different instruments of cosmetic evaluation and even with the different classifications of OS, making comparisons difficult (24). In addition, the cosmetic concept in cancer is relatively recent and, from the patient’s perspective, involves several psychosocial factors, posttreatment body acceptance, educational level, socioeconomic level and factors related to her own experience with the disease and treatment process, with the corresponding complications and sequelae.

A recent study, in which the surgeon involved in the treatment evaluated the cosmetic outcome of BCS-OS subjectively, analyzed 755 patients subjected to OS, with a mean follow-up of 74,3 months, and found 89 cosmetic sequelae. Most of these occurred during the first 3 years of follow-up; however, for major deformities classified as type III, the mean time of onset was longer. In the multivariate evaluation, postoperative complications and level II oncoplasty techniques (in which there is resection of more than 20% of the breast parenchyma, requiring remodeling and contralateral symmetrization in most cases) increased the risk of cosmetic sequelae in 4,6 and 2,6 times, respectively (22). Another study, evaluating 103 patients, found that increased BMI and breast size were associated with unsatisfactory results. A similar result was found in our study, but due to its retrospective nature, breast size was not evaluated (25).

We found different variables that negatively influenced the cosmetic outcome of classical BCS-CS and BCS-OS, both from the patient’s perspective and according to the software analysis. The time of the analysis may influence our results, as patients dissatisfied with their breasts during follow-up likely seek surgical approaches to improve the cosmetic result. In addition, when performing the OS, the surgeon has already preselected the best candidate for the procedure, typically a younger and more educated patient, which can be inferred as greater questioning of the final result of their breast reconstruction procedures. Moreover, OS is more likely to be performed for larger tumors, which requires greater resection and heavier surgical specimens to avoid mastectomy (26). These three conditions represent a bias selection associated with retrospective studies. Our analyzed population is composed of Brazilian patients assisted by the public health system, which have their particularities, requiring further studies in private patients, other centers and in other countries.

Future studies are necessary to assess the unsatisfactory results, which may help surgeons in patient selection and surgical technique. In practice it is not easy because we usually try to perform the BCS and we have many modalities associated with OS (4–7). For this condition the creation of a nomogram may anticipated unsatisfactory results, adding information for the surgeon, in order to understand whether, for the best late aesthetic result, a new surgery should be performed on the breast treated for cancer or on the contralateral one (symmetrization).

Among the limitations of the study are its retrospective and cross-sectional nature and the limited number of patients undergoing OS, which justifies the need for further longitudinal studies. In contrast, its strengths include the use of an objective, standardized and reproducible methodology (BCCT.core) associated with patient self-assessment in long-term follow-up.

## Conclusion

The different methods for evaluating cosmetic results after surgical treatment of breast cancer directly influence the identification of factors related to unsatisfactory results. Younger patients with extensive resections (large tumor size and heavy surgical specimen) and increased BMI tend to self-report evaluate the cosmetic result from OS as unsatisfactory. Understanding these results helps in sharing the decision-making process with the patient and in the training programs for breast surgeons.

## Data availability statement

The original contributions presented in the study are included in the article/**Supplementary Material**. Further inquiries can be directed to the corresponding author.

## Ethics statement

The studies involving human participants were reviewed and approved by Research Ethics Committee - Barretos Cancer Hospital. The patients/participants provided their written informed consent to participate in this study.

## Author contributions

Conceptualization and Methodology: IO-J, FS, RV, AS, EN. Funding acquisition: RV. Project administration and Supervision: EN, RV. Data curation - Patient Evaluation: FS. Data curation - BCCT.core: GB. Formal analysis: IO-J, RV, EN. Writing: IO-J, RV. Writing review & Editing: All authors. All authors contributed to the article and approved the submitted version.
