# Supplementary material for: Factors associated with unsatisfactory cosmetic results in oncoplastic surgery
Source: Front Oncol. 2023 Jul 24;13:1071127. doi: 10.3389/fonc.2023.1071127 (PMC10405917; doi:10.3389/fonc.2023.1071127)
Supplement: Supplementary file 1 [file Table_1.docx]

**Supplementary table 1.** Factors associated with unsatisfactory cosmetic results, according to the patient

|  | All cases | | | CS | | | OS | | |
| --- | --- | --- | --- | --- | --- | --- | --- | --- | --- |
| Variable | Satisfactory result N (%) | Unsatisfactory result N (%) | p | Satisfactory result N (%) | Unsatisfactory result N (%) | p | Satisfactory result N (%) | Unsatisfactory result N (%) | p |
| Age at diagnosis (years) |  |  | 0,001 |  |  | 0,029 |  |  | 0,05 |
| < 40 | 18 (7,9%) | 17 (24,6%) |  | 12 (6,8%) | 10 (20%) |  | 6 (11,3%) | 7 (36,8%) |  |
| 40 - 49 | 81 (35,4%) | 18 (26,1%) |  | 56 (31,8%) | 14 (28%) |  | 25 (47,2%) | 4 (21,1%) |  |
| 50 - 59 | 84 (36,7%) | 26 (37,7%) |  | 70 (39,8%) | 20 (40%) |  | 14 (26,4%) | 6 (31,6%) |  |
| ≥ 60 | 46 (20,1%) | 8 (11,6%) |  | 38 (21,6%) | 6 (12%) |  | 8 (15,1%) | 2 (10,5%) |  |
|  |  |  |  |  |  |  |  |  |  |
| Age at the time of evaluation (years) |  |  | 0,05 |  |  | 0,290 |  |  | 0,174 |
| < 40 | 6 (2,6%) | 5 (7,2%) |  | 3 (1,7%) | 1 (2%) |  | 3 (5,7%) | 4 (21,1%) |  |
| 40 - 49 | 24 (10,5%) | 13 (18,8%) |  | 14 (8%) | 8 (16%) |  | 10 (18,9%) | 5 (26,3%) |  |
| 50 - 59 | 97 (42,4%) | 22 (31,9%) |  | 74 (42%) | 17 (34%) |  | 23 (43,4%) | 5 (26,3%) |  |
| ≥ 60 | 102 (44,5%) | 29 (42,0%) |  | 85 (48,3%) | 24 (48%) |  | 17 (32,1%) | 5 (26,3%) |  |
|  |  |  |  |  |  |  |  |  |  |
| BMI at diagnosis (kg/m^2^) |  |  | 0,267 |  |  | 0,469 |  |  | 0,261 |
| <25 | 75 (32,8%) | 16 (23,2%) |  | 56 (31,8%) | 13 (26%) |  | 19 (35,8%) | 3 (15,8%) |  |
| 25 - 29,9 | 92 (40,2%) | 34 (49,3%) |  | 71 (40,3%) | 25 (50%) |  | 21 (39,6%) | 9 (47,4%) |  |
| ≥ 30 | 62 (27,1%) | 19 (27,5%) |  | 49 (27,8%) | 12 (24%) |  | 13 (24,5%) | 7 (36,8%) |  |
|  |  |  |  |  |  |  |  |  |  |
| BMI at the time of evaluation (kg/m^2^) |  |  | 0,137 |  |  | 0,506 |  |  | 0,03 |
| <25 | 75 (32,8%) | 14 (20,3%) |  | 56 (31,8%) | 13 (26%) |  | 19 (35,8%) | 1 (5,3%) |  |
| 25 - 29,9 | 86 (37,6%) | 32 (46,4%) |  | 68 (38,6%) | 24 (48%) |  | 18 (34%) | 8 (42,1%) |  |
| ≥ 30 | 68 (29,7%) | 23 (33,3%) |  | 52 (29,5%) | 13 (26%) |  | 10 (30,2%) | 10 (52,6%) |  |
|  |  |  |  |  |  |  |  |  |  |
| Educational level (in years) |  |  | 0,587 |  |  | 0,565 |  |  | 0,648 |
| until 8 | 142 (62%) | 47 (68,1%) |  | 117 (66,5%) | 36 (72%) |  | 25 (47,2%) | 11 (57,9%) |  |
| >8 - 11 | 48 (21%) | 11 (15,9%) |  | 33 (18,8%) | 6 (12%) |  | 15 (28,3%) | 5 (26,3%) |  |
| >11 | 39 (17%) | 11 (15,9%) |  | 26 (14,8%) | 6 (16%) |  | 13 (24,5%) | 3 (15,8%) |  |
|  |  |  |  |  |  |  |  |  |  |
| TNM stage - T |  |  | 0,013 |  |  | 0,263 |  |  | 0,022 |
| Tis and T1 | 113 (49,3%) | 23 (33,3%) |  | 91 (51,7%) | 21 (42%) |  | 22 (41,5%) | 2 (10,5%) |  |
| ≥ T2 | 116 (50,7%) | 46 (66,7%) |  | 85 (48,3%) | 29 (58%) |  | 31 (58,5%) | 17 (89,5%) |  |
|  |  |  |  |  |  |  |  |  |  |
| Tumor location |  |  | 0,906 |  |  | 0,946 |  |  | 0,463 |
| USQ | 112 (48,9%) | 32 (46,4%) |  | 95 (54%) | 26 (52%) |  | 17 (32,1%) | 6 (31,6%) |  |
| LSQ | 45 (19,7%) | 12 (17,4%) |  | 29 (16,5%) | 9 (18%) |  | 16 (30,2%) | 3 (15,8%) |  |
| MUQ | 52 (22,7%) | 17 (24,6%) |  | 40 (22,7%) | 13 (26%) |  | 12 (22,6%) | 4 (21,1%) |  |
| MLQ | 7 (3,1%) | 2 (2,9%) |  | 6 (3,4%) | 1 (2%) |  | 1 (1,9%) | 1 (5,3%) |  |
| CR | 13 (5,7%) | 6 (8,7%) |  | 6 (3,4%) | 1 (2%) |  | 7 (13,2%) | 5 (26,3%) |  |
|  |  |  |  |  |  |  |  |  |  |
|  |  |  |  |  |  |  | Continues in next | Page …….. |  |
|  |  |  |  |  |  |  |  |  |  |
|  |  |  |  |  |  |  |  |  |  |
|  |  | All cases |  |  | CS |  |  | OS |  |
| Variable | Satisfactory result N (%) | Unsatisfactory result N (%) | p | Satisfactory result N (%) | Unsatisfactory result N (%) | p | Satisfactory result N (%) | Unsatisfactory result N (%) |  |
| Tumor side |  |  | 0,891 |  |  | 0,873 |  |  | 1,000 |
| Right | 109 (47,6%) | 34 (49,3%) |  | 84 (47,7%) | 25 (50%) |  | 25 (47,2%) | 9 (47,4%) |  |
| Left | 120 (52,4%) | 35 (50,7%) |  | 92 (52,3%) | 25 (50%) |  | 28 (52,8%) | 10 (52,6%) |  |
|  |  |  |  |  |  |  |  |  |  |
| Type of axillary surgery |  |  | 0,142 |  |  | 0,089 |  |  | 1,000 |
| SLNB | 57 (24,9%) | 11 (15,9%) |  | 47 (26,7%) | 7 (14%) |  | 10 (18,9%) | 4 (21,1%) |  |
| AL | 172 (75,1%) | 58 (84,1%) |  | 129 (73,3%) | 43 (86%) |  | 43 (81,1%) | 15 (78,95) |  |
|  |  |  |  |  |  |  |  |  |  |
| Oncoplastic surgery |  |  | 0,521 |  |  |  |  |  |  |
| Present | 53 (23,1%) | 19 (27,5%) |  |  |  |  |  |  |  |
| Absent | 176 (76,9%) | 50 (72,5%) |  |  |  |  |  |  |  |
|  |  |  |  |  |  |  |  |  |  |
| Contralateral symmetrization |  |  | 1,000 |  |  |  |  |  | 0,791 |
| Present | 28 (12,2%) | 9 (13%) |  |  |  |  | 28 (52,8%) | 9 (47,4%) |  |
| Absent | 201 (87,8%) | 60 (87%) |  | 176 (100%) | 50 (50%) |  | 25 (47,2%) | 10 (52,6%) |  |
|  |  |  |  |  |  |  |  |  |  |
| Intraoperative margin widening |  |  | 0,730 |  |  | 0,695 |  |  | 1,000 |
| Present | 46 (20,1%) | 12 (17,4%) |  | 38 (21,6%) | 9 (18%) |  | 8 (15,1%) | 3 (15,8%) |  |
| Absent | 183 (79,9%) | 57 (82,6%) |  | 138 (78,4%) | 41 (82%) |  | 45 (84,9%) | 16 (84,2%) |  |
|  |  |  |  |  |  |  |  |  |  |
| Postoperative margin widening |  |  | 0,686 |  |  | 0,343 |  |  | 0,461 |
| Present | 7 (3,1%) | 1 (1,4%) |  | 6 (3,4%) | 0 |  | 1 (1,9%) | 1 (5,3%) |  |
| Absent | 222 (96,9%) | 68 (98,6%) |  | 170 (96,6%) | 50 (100%) |  | 52 (98,1%) | 18 (94,7%) |  |
|  |  |  |  |  |  |  |  |  |  |
| Surgical wound infection |  |  | 0,660 |  |  | 0,460 |  |  | 0,602 |
| Present | 26 (11,4%) | 6 (8,7%) |  | 23 (13,1%) | 4 (8,0%) |  | 3 (5,7%) | 2 (10,5%) |  |
| Absent | 203 (88,6%) | 63 (91,3%) |  | 153 (86,9%) | 46 (92%) |  | 50 (94,3%) | 17 (89,5%) |  |
|  |  |  |  |  |  |  |  |  |  |
| Dehiscence of the surgical wound |  |  | 0,323 |  |  | 0,811 |  |  | 0,280 |
| Present | 29 (12,7%) | 12 (17,4%) |  | 22 (12,5%) | 7 (14%) |  | 7 (13,2%) | 5 (26,3%) |  |
| Absent | 200 (87,3%) | 57 (82,6%) |  | 154 (87,5%) | 43 (86%) |  | 46 (86,8%) | 14 (73,7%) |  |
|  |  |  |  |  |  |  |  |  |  |
| Chemotherapy |  |  | 1,000 |  |  | 1,000 |  |  | 1,000 |
| Present | 191 (83,4%) | 58 (84,1%) |  | 145 (82,4%) | 41 (82%) |  | 46 (86,8%) | 17 (89,5%) |  |
| Absent | 38 (16,6%) | 11 (15,9%) |  | 31 (17,6%) | 9 (18%) |  | 7 (13,2%) | 2 (10,5%) |  |
|  |  |  |  |  |  |  |  |  |  |
| Hormone therapy |  |  | 0,757 |  |  | 1,000 |  |  | 0,569 |
| Present | 167 (72,9%) | 52 (75,4%) |  | 129 (73,3%) | 37 (74%) |  | 38 (71,7%) | 15 (78,9%) |  |
| Absent | 62 (27,1%) | 17 (24,6%) |  | 47 (26,7%) | 13 (26%) |  | 15 (28,3%) | 4 (21,1%) |  |
|  |  |  |  |  |  |  |  |  |  |
|  |  |  |  |  |  |  | Continues in next | Page …….. |  |
|  |  |  |  |  |  |  |  |  |  |
|  |  | All cases |  |  | CS |  |  | OS |  |
| Variable | Satisfactory result N (%) | Unsatisfactory result N (%) | p | Satisfactory result N (%) | Unsatisfactory result N (%) | p | Satisfactory result N (%) | Unsatisfactory result N (%) |  |
|  |  |  |  |  |  |  |  |  |  |
| Radiotherapy dose |  |  | 0,122 |  |  | 0,041 |  |  | 0,686 |
|  |  |  |  |  |  |  |  |  |  |
| 25 x 200 cGy | 115 (50,2%) | 25 (36,2%) |  | 83 (47,2%) | 15 (30%) |  | 32 (60,4%) | 10 (52,6%) |  |
| 28 x 180 cGy | 86 (37,6%) | 37 (53,6%) |  | 70 (39,8%) | 31 (62%) |  | 16 (30,2%) | 6 (31,6%) |  |
| 16 x 265 cGy | 14 (6,1%) | 3 (4,3%) |  | 10 (5,7%) | 1 (2%) |  | 4 (7,5%) | 2 (10,5%) |  |
| Others | 14 (6,1%) | 4 (5,8%) |  | 13 (7,4%) | 3 (6%) |  | 1 (1,9%) | 1 (5,3%) |  |
|  |  |  |  |  |  |  |  |  |  |
| Boost |  |  | 0,049 |  |  | 0,050 |  |  | 1,000 |
| Present | 198 (86,5%) | 66 (95,7%) |  | 150 (85,2%) | 48 (96%) |  | 48 (90,6%) | 18 (94,7%) |  |
| Absent | 31 (13,5%) | 3 (4,3%) |  | 26 (14,8%) | 2 (4%) |  | 5 (9,4%) | 1 (5,3%) |  |
|  |  |  |  |  |  |  |  |  |  |
| Radiotherapy SCF |  |  | 0,664 |  |  | 0,601 |  |  | 0,06 |
| Present | 76 (33,5%) | 21 (30,4%) |  | 51 (29,3%) | 17 (34%) |  | 25 (47,2%) | 4 (21,1%) |  |
| Absent | 151 (66,5%) | 48 (69,6%) |  | 123 (79,7%) | 33 (66%) |  | 28 (52,8%) | 15 (78,9%) |  |
|  |  |  |  |  |  |  |  |  |  |
| Lymphedema |  |  | 0,744 |  |  | 1,000 |  |  | 0,531 |
| Present | 51 (22,3%) | 14 (20,3%) |  | 37 (21%) | 11 (22%) |  | 14 (26,4%) | 3 (15,8%) |  |
| Absent | 178 (77,7%) | 55 (79,7%) |  | 139 (79%) | 39 (78%) |  | 39 (73,6%) | 16 (84,2%) |  |
|  |  |  |  |  |  |  |  |  |  |
| Radiodermatitis at the end of radiotherapy |  |  | 0,826 |  |  | 1,000 |  |  | 0,288 |
| Absent | 9 (3,9%) | 2 (2,9%) |  | 7 (4%) | 2 (4%) |  | 2 (3,8%) | 0 |  |
| Grade 1 | 128 (55,9%) | 35 (50,7%) |  | 95 (54%) | 27 (54%) |  | 33 (62,3%) | 8 (42,1%) |  |
| Grade 2 | 74 (32,3%) | 25 (36,2%) |  | 62 (35,2%) | 17 (34%) |  | 12 (22,6%) | 8 (42,1%) |  |
| Grade 3 | 18 (7,9%) | 7 (10,1%) |  | 12 (6,8%) | 4 (8%) |  | 6 (11,3%) | 3 (15,8%) |  |

CS - classical surgery/ OS - oncoplastic surgery/ BMI - body mass index/ USQ - upper side quadrant/ LSQ - lower side quadrant/ MUQ - medial upper quadrant/ MLQ - medial lower quadrant/ CR - central region/ SLNB - sentinel lymph node biopsy/ AL - axillary lymphadenectomy/ SCF - supraclavicular fossa

**Supplementary table 2.** Factors associated with unsatisfactory cosmetic results, in the patient's view, according to the surgery performed, numerically

|  | **All cases** | | | | | | | **CS** | | | | | | | **OS** | | | | | | |
| --- | --- | --- | --- | --- | --- | --- | --- | --- | --- | --- | --- | --- | --- | --- | --- | --- | --- | --- | --- | --- | --- |
| **Variable** | **Satisfactory result** | | | **Unsatisfactory result** | | | **p** | **Satisfactory result** | | | **Unsatisfactory result** | | | **p** | **Satisfactory result** | | | **Unsatisfactory result** | | | **p** |
|  | Mean | Median | SD | Mean | Median | SD |  | Mean | Median | SD | Mean | Median | SD |  | Mean | Median | SD | Mean | Median | SD |  |
| **Age at diagnosis (years)** | 51,8 | 51 | 9,48 | 47,8 | 49 | 10,67 | 0,003 | 52,4 | 52,0 | 9,28 | 49,1 | 50,0 | 9,98 | 0,04 | 49,9 | 47,0 | 9,9 | 44,4 | 45,0 | 11,9 | 0,158 |
|  |  |  |  |  |  |  |  |  |  |  |  |  |  |  |  |  |  |  |  |  |  |
| **Age at the time of evaluation (years)** | 59,36 | 59,04 | 9,22 | 56,25 | 57,95 | 10,75 | 0,001 | 60,3 | 59,8 | 9,0 | 58,3 | 59,4 | 9,6 | 0,347 | 56,2 | 55,2 | 9,2 | 50,7 | 50,2 | 11,7 | 0,079 |
|  |  |  |  |  |  |  |  |  |  |  |  |  |  |  |  |  |  |  |  |  |  |
| **BMI at diagnosis (kg/m^2^)** | 27,6 | 26,7 | 4,9 | 27,5 | 26,7 | 4,5 | 0,764 | 27,9 | 26,8 | 5,5 | 27,0 | 26,5 | 4,1 | 0,63 | 27,3 | 26,3 | 5,3 | 28,7 | 26,8 | 5,3 | 0,210 |
|  |  |  |  |  |  |  |  |  |  |  |  |  |  |  |  |  |  |  |  |  |  |
| **BMI at the time of evaluation (kg/m^2^)** | 28,24 | 27,34 | 5,38 | 28,6 | 28,54 | 4,97 | 0,186 | 28,5 | 27,5 | 5,5 | 27,5 | 28,1 | 4,4 | 0,804 | 27,4 | 27,1 | 4,8 | 31,7 | 31,1 | 5,1 | 0,002 |
|  |  |  |  |  |  |  |  |  |  |  |  |  |  |  |  |  |  |  |  |  |  |
| **Tumor size (cm)** | 2,34 | 2 | 1,5 | 2,63 | 2,5 | 1,4 | 0,04 | 2,1 | 2,0 | 1,1 | 2,4 | 2,1 | 1,4 | 0,483 | 2,9 | 2,5 | 2,1 | 3,3 | 3,0 | 1,1 | 0,03 |
|  |  |  |  |  |  |  |  |  |  |  |  |  |  |  |  |  |  |  |  |  |  |
| **Weight of the surgical specimen (g)** | 143,9 | 113 | 112,49 | 170,85 | 120 | 119,01 | 0,123 | 132,1 | 110,0 | 100,5 | 147,3 | 100,0 | 106,6 | 0,664 | 183,0 | 142,0 | 139,5 | 232,9 | 202,0 | 130,1 | 0,05 |
|  |  |  |  |  |  |  |  |  |  |  |  |  |  |  |  |  |  |  |  |  |  |
| **Distance from the surgical margin (cm)** | 0,93 | 0,9 | 0,6 | 0,96 | 1,0 | 0,57 | 0,590 | 0,89 | 0,80 | 0,58 | 0,93 | 1,0 | 0,53 | 0,467 | 1,1 | 1,0 | 0,64 | 1,0 | 1,0 | 0,68 | 0,753 |
|  |  |  |  |  |  |  |  |  |  |  |  |  |  |  |  |  |  |  |  |  |  |
| **Time between surgery and evaluation (years)** | 6,66 | 5,88 | 4,25 | 7,51 | 7,47 | 3,71 | 0,035 | 6,8 | 6,3 | 4,3 | 8,0 | 8,7 | 3,6 | 0,019 | 6,0 | 4,7 | 4,1 | 6,2 | 5,1 | 3,7 | 0,578 |
|  |  |  |  |  |  |  |  |  |  |  |  |  |  |  |  |  |  |  |  |  |  |
| **Time between the end of the RT and the evaluation (years)** | 6,05 | 5,03 | 4,32 | 7 | 6,75 | 3,84 | 0,028 | 6,3 | 5,5 | 4,3 | 7,5 | 7,9 | 3,7 | 0,018 | 5,2 | 3,9 | 4,1 | 5,6 | 4,4 | 3,8 | 0,527 |

CS - classical surgery/ OS - oncoplastic surgery/ BMI - body mass index/ RT - radiotherapy/ SD - standard deviation

**Supplementary table 3.** Factors associated with unsatisfactory cosmetic results, according to the BCCT.core software

|  | All cases | | | CS | | | OS | | |
| --- | --- | --- | --- | --- | --- | --- | --- | --- | --- |
| Variable | Satisfactory result N(%) | Unsatisfactory result N (%) | p | Satisfactory result N (%) | Unsatisfactory result N (%) | p | Satisfactory result N (%) | Unsatisfactory result N (%) | p |
| Age at diagnosis (years) |  |  | 0,483 |  |  | 0,530 |  |  | 0,726 |
| < 40 | 12 (13,5%) | 24 (11,5%) |  | 7 (10,3%) | 16 (10,2%) |  | 5 (23,8%) | 8 (15,7%) |  |
| 40 - 49 | 28 (31,5%) | 70 (33,7%) |  | 19 (27,9%) | 50 (31,8%) |  | 9 (42,9%) | 20 (39,2%) |  |
| 50 - 59 | 29 (32,6%) | 81 (38,9%) |  | 25 (36,8%) | 65 (41,4%) |  | 4 (19%) | 16 (31,4%) |  |
| ≥ 60 | 20 (22,5%) | 33 (15,9%) |  | 17 (25%) | 26 (16,6%) |  | 3 (14,3%) | 7 (13,7%) |  |
|  |  |  |  |  |  |  |  |  |  |
| Age at the time of evaluation (years) |  |  | 0,185 |  |  | 0,186 |  |  | 0,506 |
| < 40 | 6 (6,7%) | 5 (2,4%) |  | 3 (4,4%) | 1 (0,6%) |  | 3 (14,3%) | 4 (7,8%) |  |
| 40 - 49 | 11 (12,4%) | 27 (13%) |  | 6 (8,8%) | 17 (10,8%) |  | 5 (23,8%) | 10 (19,6%) |  |
| 50 - 59 | 39 (43,8%) | 80 (38,5%) |  | 30 (44,1%) | 61 (38,9%) |  | 9 (42,9%) | 19 (37,3%) |  |
| ≥ 60 | 33 (37,1%) | 96 (46,2%) |  | 29 (42,6%) | 78 (49,7%) |  | 4 (19%) | 18 (35,3%) |  |
|  |  |  |  |  |  |  |  |  |  |
| BMI at diagnosis (kg/m^2^) |  |  | 0,002 |  |  | 0,007 |  |  | 0,278 |
| <25 | 40 (44,9%) | 52 (25,0%) |  | 31 (45,6%) | 39 (24,8%) |  | 9 (42,9%) | 13 (25,5%) |  |
| 25 - 29,9 | 27 (30,3%) | 98 (47,1%) |  | 21 (30,9%) | 74 (47,1%) |  | 6 (28,6%) | 24 (47,1%) |  |
| ≥ 30 | 22 (24,7%) | 58 (27,9%) |  | 16 (23,5%) | 44 (28,0%) |  | 6 (28,6%) | 14 (27,5%) |  |
|  |  |  |  |  |  |  |  |  |  |
| BMI at the time of evaluation (kg/m^2^) |  |  | 0,023 |  |  | 0,065 |  |  | 0,298 |
| <25 | 36 (40,4%) | 52 (25%) |  | 28 (41,2%) | 40 (25,5%) |  | 8 (38,1%) | 12 (23,5%) |  |
| 25 - 29,9 | 32 (36%) | 86 (41,3%) |  | 24 (35,3%) | 68 (43,3%) |  | 8 (38,1%) | 18 (35,3%) |  |
| ≥ 30 | 21 (23,6%) | 70 (33,7%) |  | 16 (23,5%) | 49 (31,2%) |  | 5 (23,8%) | 21 (41,2%) |  |
|  |  |  |  |  |  |  |  |  |  |
| Educational level (in years) |  |  | 0,143 |  |  | 0,038 |  |  | 0,837 |
| until 8 | 50 (56,2%) | 137 (65,9%) |  | 40 (58,8%) | 111 (70,7%) |  | 10 (47,6%) | 26 (51%) |  |
| >8 - 11 | 18 (20,2%) | 41 (19,7%) |  | 11 (16,2%) | 28 (17,8%) |  | 7 (33,3%) | 13 (25,5%) |  |
| >11 | 21 (23,6%) | 30 (14,4%) |  | 17 (25%) | 18 (11,5%) |  | 4 (19%) | 12 (23,5%) |  |
|  |  |  |  |  |  |  |  |  |  |
| TNM stage - T |  |  | 0,05 |  |  | 0,192 |  |  | 0,168 |
| Tis and T1 | 48 (53,9%) | 86 (41,3%) |  | 38 (55,9%) | 72 (45,9%) |  | 10 (47,6%) | 14 (27,5%) |  |
| ≥ T2 | 41 (46,1%) | 122 (58,7%) |  | 30 (44,1%) | 85 (54,1%) |  | 11 (52,4%) | 37 (72,5%) |  |
|  |  |  |  |  |  |  |  |  |  |
| Tumor location |  |  | 0,084 |  |  | 0,392 |  |  | 0,185 |
| USQ | 40 (44,9%) | 104 (50%) |  | 34 (50%) | 87 (55,4%) |  | 6 (28,6%) | 17 (33,3%) |  |
| LSQ | 21 (23,6%) | 36 (17,3%) |  | 12 (17,6%) | 26 (16,6%) |  | 9 (42,9%) | 10 (19,6%) |  |
| MUQ | 25 (28,1%) | 44 (21,2%) |  | 20 (29,4%) | 33 (21%) |  | 5 (23,8%) | 11 (21,6%) |  |
| MLQ | 0 | 8 (3,8%) |  | 0 | 6 (3,8%) |  | 0 | 2 (3,9%) |  |
| CR | 3 (3,4%) | 16 (7,7%) |  | 2 (2,9%) | 5 (3,2%) |  | 1 (4,8%) | 11 (21,6%) |  |
|  |  |  |  |  |  |  |  |  |  |
|  |  |  |  |  |  |  | Continues in next | Page …….. |  |
|  |  |  |  |  |  |  |  |  |  |
|  |  |  |  |  |  |  |  |  |  |
|  |  | **All cases** |  |  | **CS** |  |  | **OS** |  |
| Variable | Satisfactory result N(%) | Unsatisfactory result N (%) | p | Satisfactory result N (%) | Unsatisfactory result N (%) | p | Satisfactory result N (%) | Unsatisfactory result N (%) | p |
| Tumor side |  |  | 0,002 |  |  | 0,021 |  |  | 0,041 |
| Right | 55 (61,8%) | 88 (42,3%) |  | 41 (60,3%) | 68 (43,3%) |  | 14 (66,7%) | 20 (39,2%) |  |
| Left | 34 (38,2%) | 120 (57,7%) |  | 27 (39,7%) | 89 (56,7%) |  | 7 (33,3%) | 31 (60,8%) |  |
|  |  |  |  |  |  |  |  |  |  |
| Type of axillary surgery |  |  | 0,001 |  |  | 0,007 |  |  | 0,098 |
| SLNB | 32 (36%) | 37 (17,8%) |  | 25 (36,8%) | 30 (19,1%) |  | 7 (33,3%) | 7 (13,7%) |  |
| AL | 57 (64%) | 171 (82,2%) |  | 43 (63,25) | 127 (80,9%) |  | 14 (66,7%) | 44 (86,3%) |  |
|  |  |  |  |  |  |  |  |  |  |
| Oncoplastic surgery |  |  | 0,884 |  |  |  |  |  |  |
| Present | 21 (23,6%) | 51 (24,5%) |  |  |  |  |  |  |  |
| Absent | 68 (76,4%) | 157 (75,5%) |  |  |  |  |  |  |  |
|  |  |  |  |  |  |  |  |  |  |
| Contralateral symmetrization |  |  | 0,337 |  |  |  |  |  | 0,791 |
| Present | 14 (15,7%) | 23 (11,1%) |  |  |  |  | 28 (52,8%) | 9 (47,4%) |  |
| Absent | 75 (84,3%) | 185 (88,9%) |  | 68 (100%) | 157 (100%) |  | 25 (47,2%) | 10 (52,6%) |  |
|  |  |  |  |  |  |  |  |  |  |
| Intraoperative margin widening |  |  | 0,874 |  |  | 0,858 |  |  | 1,000 |
| Present | 18 (20,2%) | 40 (19,2%) |  | 15 (22,1%) | 32 (20,4%) |  | 3 (14,3%) | 8 (15,7%) |  |
| Absent | 71 (79,8%) | 168 (80,8%) |  | 53 (77,9%) | 125 (79,6%) |  | 18 (85,7%) | 43 (84,3%) |  |
|  |  |  |  |  |  |  |  |  |  |
| Postoperative margin widening |  |  | 1,000 |  |  | 1,000 |  |  | 1,000 |
| Present | 2 (2,2%) | 6 (2,9%) |  | 2 (2,9%) | 4 (2,5%) |  | 0 | 2 (3,9%) |  |
| Absent | 87 (97,8%) | 202 (97,1%) |  | 66 (97,1%) | 153 (97,5% |  | 21 (100%) | 49 (96,1%) |  |
|  |  |  |  |  |  |  |  |  |  |
| Surgical wound infection |  |  | 0,273 |  |  | 0,521 |  |  | 0,489 |
| Present | 9 (10,1%) | 32 (15,4%) |  | 7 (10,3%) | 22 (14%) |  | 2 (9,5%) | 10 (19,6%) |  |
| Absent | 80 (89,9%) | 176 (84,6%) |  | 61 (89,7%) | 135 (86%) |  | 19 (90,5%) | 41 (80,4%) |  |
|  |  |  |  |  |  |  |  |  |  |
| Dehiscence of the surgical wound |  |  | 0,067 |  |  | 0,075 |  |  | 1,000 |
| Present | 5 (5,6%) | 27 (13%) |  | 4 (5,9%) | 23 (14,6%) |  | 1 (4,8%) | 4 (7,8%) |  |
| Absent | 84 (94,4%) | 181 (87%) |  | 64 (94,1%) | 134 (85,4%) |  | 20 (95,2%) | 47 (92,2%) |  |
|  |  |  |  |  |  |  |  |  |  |
| Chemotherapy |  |  | 0,006 |  |  | 0,036 |  |  | 0,111 |
| Present | 66 (74,2%) | 182 (87,5%) |  | 50 (73,5%) | 135 (86%) |  | 16 (76,2%) | 47 (92,2%) |  |
| Absent | 23 (25,8%) | 26 (12,5%) |  | 18 (26,5%) | 22 (14%) |  | 5 (23,8%) | 4 (7,8%) |  |
|  |  |  |  |  |  |  |  |  |  |
|  |  |  |  |  |  |  | Continues in next | Page …….. |  |
|  |  |  |  |  |  |  |  |  |  |
|  |  |  |  |  |  |  |  |  |  |
|  |  |  |  |  |  |  |  |  |  |
|  |  | **All cases** |  |  | **CS** |  |  | **OS** |  |
| Variable | Satisfactory result N(%) | Unsatisfactory result N (%) | p | Satisfactory result N (%) | Unsatisfactory result N (%) | p | Satisfactory result N (%) | Unsatisfactory result N (%) | p |
| Hormone therapy |  |  | 0,151 |  |  | 0,252 |  |  | 0,400 |
| Present | 71 (79,8%) | 149 (71,6%) |  | 54 (79,4%) | 113 (72%) |  | 17 (81%) | 36 (70,6%) |  |
| Absent | 18 (20,2%) | 59 (28,4%) |  | 14 (20,6%) | 44 (28%) |  | 4 (19%) | 15 (29,4%) |  |
|  |  |  |  |  |  |  |  |  |  |
| Radiotherapy dose |  |  | 0,004 |  |  | 0,001 |  |  | 0,893 |
| 25 x 200 cGy | 49 (55,1%) | 92 (44,2%) |  | 37 (54,4%) | 62 (39,5%) |  | 12 (57,1%) | 30 (58,8%) |  |
| 28 x 180 cGy | 24 (27%) | 98 (47,1%) |  | 18 (26,5%) | 82 (52,2%) |  | 6 (28,6%) | 16 (31,4%) |  |
| 16 x 265 cGy | 9 (10,1%) | 8 (3,8%) |  | 7 (10,3%) | 4 (2,5%) |  | 2 (9,5%) | 4 (7,8%) |  |
| Others | 7 (7,9%) | 10 (4,8%) |  | 6 (8,8%) | 9 (5,7%) |  | 1 (4,8%) | 1 (2%) |  |
|  |  |  |  |  |  |  |  |  |  |
| Boost |  |  | 1,000 |  |  | 0,828 |  |  | 0,664 |
| Present | 79 (88,8%) | 184 (88,5%) |  | 59 (86,8%) | 138 (87,9%) |  | 20 (95,2%) | 46 (90,2%) |  |
| Absent | 10 (11,2%) | 24 (11,5%) |  | 9 (13,2%) | 19 (12,1%) |  | 1 (4,8%) | 5 (9,8%) |  |
|  |  |  |  |  |  |  |  |  |  |
| Radiotherapy SCF |  |  | 0,787 |  |  | 0,751 |  |  | 0,197 |
| Present | 30 (34,5%) | 68 (32,7%) |  | 19 (28,8%) | 50 (31,8%) |  | 11 (52,4%) | 18 (35,3%) |  |
| Absent | 57 (65,5%) | 140 (67,3%) |  | 47 (71,2%) | 107 (68,2%) |  | 10 (47,6%) | 33 (64,7%) |  |
|  |  |  |  |  |  |  |  |  |  |
| Lymphedema |  |  | 0,031 |  |  | 0,116 |  |  | 0,125 |
| Present | 12 (13,5%) | 53 (25,5%) |  | 10 (14,7%) | 38 (24,2%) |  | 2 (9,5%) | 15 (29,4%) |  |
| Absent | 77 (86,5%) | 155 (74,5%) |  | 58 (85,3%) | 119 (75,8%) |  | 19 (90,5%) | 36 (70,6%) |  |
|  |  |  |  |  |  |  |  |  |  |
| Radiodermatitis at the end of radiotherapy |  |  | 0,758 |  |  | 0,929 |  |  | 0,288 |
| Absent | 4 (4,5%) | 7 (3,4%) |  | 2 (2,9%) | 7 (4,5%) |  | 2 (3,8%) | 0 |  |
| Grade 1 | 50 (56,2%) | 112 (53,8%) |  | 38 (55,9%) | 83 (52,9%) |  | 33 (62,3%) | 8 (42,1%) |  |
| Grade 2 | 30 (33,7%) | 70 (33,7%) |  | 24 (35,3%) | 56 (35,7%) |  | 12 (22,6%) | 8 (42,1%) |  |
| Grade 3 | 5 (5,6%) | 19 (9,1%) |  | 4 (5,9%) | 11 (7%) |  | 6 (11,3%) | 3 (15,8%) |  |

CS - classical surgery/ OS - oncoplastic surgery/ BMI - body mass index/ USQ - upper side quadrant/ LSQ - lower side quadrant/ MUQ - medial upper quadrant/ MLQ - medial lower quadrant/ CR - central region/ SLNB - sentinel lymph node biopsy/ AL - axillary lymphadenectomy/ SCF - supraclavicular fossa

**Supplementary table 4.** Factors associated with unsatisfactory cosmetic results, in the BCCT.core software evaluation, according to the surgery performed, numerically

|  | **All cases** | | | | | | | **CS** | | | | | | | | **OS** | | | | | | | |  |
| --- | --- | --- | --- | --- | --- | --- | --- | --- | --- | --- | --- | --- | --- | --- | --- | --- | --- | --- | --- | --- | --- | --- | --- | --- |
| **Variable** | **Satisfactory result** | | | **Unsatisfactory result** | | | **p** | | **Satisfactory result** | | | **Unsatisfactory result** | | | **p** | | **Satisfactory result** | | | **Unsatisfactory result** | | | **p** | |
|  | Mean | Median | SD | Mean | Median | SD |  | | Mean | Median | SD | Mean | Median | SD |  | | Mean | Median | SD | Mean | Median | SD |  | |
| **Age at diagnosis (years)** | 51,1 | 51 | 11 | 50,6 | 51 | 9,3 | 0,67 | | 52,4 | 52 | 10,2 | 51,1 | 52,0 | 9,18 | 0,424 | | 47,0 | 46,0 | 12,9 | 48,9 | 48,0 | 9,7 | 0,340 | |
|  |  |  |  |  |  |  |  | |  |  |  |  |  |  |  | |  |  |  |  |  |  |  | |
| **Age at the time of evaluation (years)** | 56,8 | 55,8 | 10,5 | 59,2 | 59,4 | 9,1 | 0,06 | | 58,2 | 57,8 | 10,1 | 60,3 | 59,9 | 8,7 | 0,089 | | 52,4 | 52,2 | 11,0 | 55,8 | 55,2 | 9,7 | 0,252 | |
|  |  |  |  |  |  |  |  | |  |  |  |  |  |  |  | |  |  |  |  |  |  |  | |
| **BMI at diagnosis (kg/m^2^)** | 26,8 | 25,2 | 5,4 | 27,9 | 26,9 | 4,6 | 0,011 | | 26,6 | 25,1 | 5,0 | 28,1 | 27,0 | 5,2 | 0,017 | | 27,4 | 26,2 | 6,4 | 27,8 | 26,7 | 4,8 | 0,376 | |
|  |  |  |  |  |  |  |  | |  |  |  |  |  |  |  | |  |  |  |  |  |  |  | |
| **BMI at the time of evaluation (kg/m^2^)** | 26,9 | 25,7 | 5,0 | 28,9 | 28,2 | 5,27 | 0,001 | | 26,8 | 25,6 | 4,8 | 28,9 | 28,3 | 5,3 | 0,001 | | 27,6 | 27,3 | 5,7 | 28,9 | 28,0 | 5,1 | 0,287 | |
|  |  |  |  |  |  |  |  | |  |  |  |  |  |  |  | |  |  |  |  |  |  |  | |
| **Tumor size (cm)** | 2,2 | 2,0 | 1,2 | 2,5 | 2,2 | 1,5 | 0,11 | | 2,1 | 1,8 | 1,1 | 2,2 | 2,0 | 1,26 | 0,420 | | 2,5 | 2,3 | 1,3 | 3,3 | 2,8 | 2,1 | 0,115 | |
|  |  |  |  |  |  |  |  | |  |  |  |  |  |  |  | |  |  |  |  |  |  |  | |
| **Weight of the surgical specimen (g)** | 117,2 | 86 | 92,8 | 164,6 | 132,5 | 120,1 | < 0,001 | | 94,8 | 77,5 | 53,9 | 153,5 | 115,0 | 112,6 | < 0,001 | | 189,8 | 136,0 | 144,5 | 198,8 | 156,0 | 136,6 | 0,569 | |
|  |  |  |  |  |  |  |  | |  |  |  |  |  |  |  | |  |  |  |  |  |  |  | |
| **Distance from the surgical margin (cm)** | 0,82 | 0,8 | 0,53 | 0,99 | 1,0 | 0,61 | 0,01 | | 0,77 | 0,70 | 0,48 | 0,95 | 1,0 | 0,59 | 0,018 | | 1,0 | 1,0 | 0,65 | 1,1 | 1,0 | 0,65 | 0,554 | |
|  |  |  |  |  |  |  |  | |  |  |  |  |  |  |  | |  |  |  |  |  |  |  | |
| **Time between surgery and evaluation (years)** | 5,1 | 4,4 | 3,1 | 7,5 | 7,3 | 4,3 | < 0,001 | | 5,1 | 4,4 | 3,1 | 7,9 | 7,8 | 4,3 | < 0,001 | | 5,3 | 4,7 | 3,6 | 6,3 | 5,0 | 4,1 | 0,307 | |
|  |  |  |  |  |  |  |  | |  |  |  |  |  |  |  | |  |  |  |  |  |  |  | |
| **Time between the end of the RT and the evaluation (years)** | 4,5 | 3,7 | 3,1 | 6,9 | 6,5 | 4,4 | < 0,001 | | 4,5 | 3,6 | 3,1 | 7,4 | 7,3 | 4,4 | < 0,001 | | 4,7 | 3,9 | 3,6 | 5,5 | 4,4 | 4,2 | 0,461 | |

CS - classical surgery/ OS - oncoplastic surgery/ BMI - body mass index/ RT - radiotherapy/ SD - standard deviation
